# Growth-promoting hormonal alterations in pregnant women living with HIV receiving dolutegravir-based antiretroviral treatment are associated with lower infant 1-year weight z-scores

**DOI:** 10.3389/fped.2025.1559877

**Published:** 2025-07-25

**Authors:** Kathleen M. Powis, Jennifer Jao, Justine Legbedze, Caroline Dunk, Keolebogile N. Mmasa, Samuel W. Kgole, Gosego Masasa, Terence Mohammed, Joseph Makhema, Sikhulile Moyo, Mitchell E. Geffner, Elaine J. Abrams, Lena Serghides

**Affiliations:** ^1^ Departments of Internal Medicine and Pediatrics, Massachusetts General Hospital, Boston, MA, United States; ^2^ Department of Immunology and Infectious Diseases, Harvard T.H. Chan School of Public Health, Boston, MA, United States; ^3^ Botswana Harvard Health Partnership, Gaborone, Botswana; ^4^ Ann and Robert H. Lurie Children's Hospital of Chicago, Chicago, IL, United States, Department of Pediatrics; ^5^ Feinberg School of Medicine, Department of Pediatrics, Division of Infectious Diseases, Department of Medicine, Division of Adult Infectious Diseases, Northwestern University, Chicago, IL, United States; ^6^ Toronto General Hospital Research Institute, University Health Network, Toronto, ON, Canada; ^7^ County Durham and Darlington NHS Foundation Trust, Darlington Memorial Hospital, Darlington, United Kingdom; ^8^ Keck School of Medicine of the University of Southern California, The Saban Research Institute of Children's Hospital Los Angeles, Los Angeles, CA, United States; ^9^ Department of Pediatrics, ICAP at Columbia University, Vagelos College of Physicians & Surgeons, Columbia University, New York, NY, United States; ^10^ Department of Immunology and Institute of Medical Sciences, University of Toronto, Toronto, ON, Canada

**Keywords:** HIV, pregnancy, growth hormone, infants HIV exposed uninfected, infant anthropometrics

## Abstract

**Background:**

Several maternal hormones regulate fetal growth, but concentrations of these hormones in pregnancy among women living with HIV and associations between hormone levels and infant anthropometrics is limited.

**Methods:**

Pregnant women with HIV receiving dolutegravir/tenofovir/emtricitabine and HIV-seronegative women and their infants prospectively enrolled in the Botswana-based Tshilo Dikotla study were included in this analysis. Estradiol, sex-hormone binding globulin (SHBG), progesterone, cortisol, pituitary growth hormone-1 (GH1), insulin-like growth factor-1 (IGF-1), and insulin-like growth factor binding protein-1 (IGFBP-1) concentrations were measured in maternal plasma collected between 24 and 29 weeks’ gestation. Bioavailable estradiol was derived using estradiol and SHBG concentrations. Generalized linear models were fit to evaluate associations between HIV status and each maternal hormone. Similar models were fit to assess effect modification by HIV status on the relationship between each maternal hormone and infant anthropometrics at birth and 1-year of age.

**Results:**

Maternal plasma specimens were available from 114 women (46 with HIV). Women with HIV had lower mean log bioavailable estradiol (β: −0.22, *p* = 0.031), cortisol (β: −0.22, *p* = 0.001), and IGF-1 (β: −0.81, *p* = 0.007), but higher GH1 (β: 0.91, *p* = 0.007) than women without HIV. Infant HIV-exposure status modified associations of log GH1 (β: −0.21, *p* = 0.05) and log IGF-1 (β: 0.40, *p* = 0.004) with infant 1-year weight-for-age z-score (WAZ), adjusting for maternal age, BMI, exclusive breastfeeding duration, and birth WAZ. Among infants who were HIV exposed uninfected, lower GH1 and higher IGF1 levels were associated with higher WAZ at 1 year of age. These associations were not observed in HIV-unexposed infants.

**Conclusion:**

Associations between maternal growth-promoting hormones and infant weight at 1 year of life differ significantly by maternal HIV status, reflecting potential perturbations in the maternal-fetal-infant growth axis among pregnant women with HIV. Additional research is needed to identify mechanisms and possible interventions.

## 1 Introduction

Successful global implementation of a public health policy promoting universal access to antiretroviral treatment (ART) at time of HIV diagnoses for all pregnant and postpartum women living with HIV has substantially reduced infant HIV acquisition, resulting in a population in 2022 of approximately 16 million children ≤ 14 years of age born HIV-exposed who have remained uninfected (1). Yet, starting life HIV-free falls short of ensuring the health and well-being of all children with perinatal exposure to HIV, and in many cases, maternal ART. Studies have demonstrated that children who are HIV-exposed but remain uninfected (HEU) experience higher risk of infectious morbidity and mortality, poorer growth, metabolic perturbations, and neurodevelopmental challenges (2–10). While these disparities have been associated with postnatal factors, including maternal physical and mental health, maternal education, household food insecurity, poor access to clean water and sanitation, and other sociodemographic risk factors (11, 12), disturbances in biologic pathways during gestation may also contribute to poorer growth outcomes in this population (6, 10, 13).

In a healthy pregnancy, fetal growth is tightly regulated by maternal and placental hormones and growth factors that, in turn, regulate fetal hormones and growth factors responsible for fetal weight gain and linear growth (14–21). The placenta, serving as an interface between the mother and fetus, mediates exchange of nutrients responsible for fetal growth (22). By 15–17 weeks gestation, placental production of growth hormone (GH2) should rise, suppressing production of pituitary derived growth hormone (GH1), with GH2 levels peaking at the conclusion of the pregnancy (21, 22). GH2, in combination with maternal progesterone, cortisol, inflammatory cytokines and other hormones, increases maternal insulin resistance and lipolysis in the second half of pregnancy, freeing maternal glucose, amino acids, essential fatty acids, and ketones for transport to the fetus (21). Concomitantly, GH2 induces increased production of maternal estrogen, insulin-like growth factor-1 (IGF-1) and insulin-like growth factor-2 (IGF-2), which jointly promote uterine growth and blood flow necessary for optimal transplacental nutrient delivery and fetal growth (21, 23). Hormones in pregnancy have been found to be altered in pregnant women with HIV (24–27). While the maternal-fetal-infant growth axis is clearly complex, identification of alterations in maternal hormones and/or growth factors associated with fetal and postnatal growth among pregnant women with HIV may provide a modifiable biological mechanism by which birth outcomes and infant growth could be improved for infants who are HEU, particularly those with low weight or length at birth. An important potential mechanism warranting investigation is the ART regimen used during pregnancy. Currently, the World Health Organization recommends use of an integrase strand transfer inhibitor (INSTI)-based regimen as a first line regimen in pregnancy, with the preferred INSTI being dolutegravir (28). Here we compared levels of maternal systemic hormones, growth factors, and relevant binding proteins between pregnant women living with HIV and receiving a dolutegravir containing ART regimen and pregnant women who were HIV-seronegative. In addition, we evaluated the relationship between these analytes and infant weight and length z-scores at birth and 1 year of life.

## 2 Materials and methods

### Study population

The Tshilo Dikotla study enrolled pregnant women, both those with HIV and those HIV-seronegative. Eligible participants had to have expressed an intention to breastfeed their infant, were ≥18 years of age, and were between 16 and 36 weeks gestational age (GA) at enrollment. Participants were enrolled between August 2016 - May 2019, in Gaborone, Botswana. Other inclusion and exclusion criteria have previously been described (NCT03088410) (29). Briefly, pregnant women with pre-existing diabetes or multi-fetal gestations were excluded. For this analysis, pregnant women with HIV receiving tenofovir disoproxil fumarate (TDF)/emtricitabine or lamivudine (XTC) plus dolutegravir (DTG) were included. This analysis focused on participants with a plasma specimen drawn between 24- and 29-weeks gestation, as this is the period in gestation where maternal and placental hormones and growth factors prime exponential fetal growth (20, 21). Additionally, previous studies have reported significant associations between progesterone levels at this gestational window and infant birth weight born to women with HIV on protease inhibitor-based ART (24). Pregnant women were enrolled as HIV-seronegative if they had documentation of a negative HIV test in pregnancy or tested negative at time of study enrollment and between 32 weeks and delivery to ensure absence of seroconversion. For HIV-seronegative women who were breastfeeding, HIV testing was performed at each study visit until six weeks after breastfeeding cessation.

Among infants born to women with HIV, HIV DNA PCR was performed at birth and one month of life and at all subsequent study visits if infants were still breastfeeding, including at least six weeks after breastfeeding cessation. HIV testing via ELISA was also performed at 18 months of life.

### Ethics statement

This study was approved by the Institutional Review Boards at the Health Research and Development Committee in Botswana, Ann and Robert H. Lurie Children's Hospital of Chicago, Massachusetts General Hospital, and University Health Network. Adults provided informed consent for their own and their infant's participation.

### Primary outcomes

The primary outcomes of interest included maternal systemic levels of bioavailable estradiol, cortisol, sex hormone binding globulin (SHBG), progesterone, pituitary growth hormone-1 (GH1), insulin-like growth factor-1 (IGF-1), and insulin-line growth factor binding protein-1 (IGFBP-1) between 24- and 29-weeks' gestation, and infant weight-for-age (WAZ) and length-for-age (LAZ) z-scores at birth and one-year of life. All analytes were assayed by enzyme-linked immunosorbent assay (ELISA) according to the manufacturers' instructions on maternal plasma collected as morning specimens (between 7 AM and 12 PM). Estradiol, SHBG, and cortisol kits were from DRG International (Springfield, NJ, USA). GH1, IGF1, and IGFBP-1 kits were from R&D Systems (Minneapolis, MN, USA). The IGF1 assays measured free IGF-1 (unbound). Progesterone kits were from ENZO Biochem (Farmingdale, NY, USA). EDTA plasma samples were diluted 1:50 for estradiol, 1:300 for SHBG, 1:2 for cortisol, 1:1,000 for progesterone, 1:5 for GH1, 1:2 for IGF1, and 1:300 for IGFBP1. All samples were assayed in duplicate. Bioavailable estradiol was calculated using a simplified 1-ligand/2-protein version of the method published by Mazer (30), using measured estradiol (ligand) and SHBG (protein) and assumed a default value of 4.3 g/dl for albumin (protein).

Infant birth weight and length were abstracted from the infant's medical record. Study staff evaluated infant weight and length at the 1-year Tshilo Dikotla study visit. A calibrated infant scale was used to assess weight, using an algorithm of three sequential measurements. Infant clothing was removed, including the infant's diaper, prior to obtaining the weight. Infant length was obtained by two trained study staff members, using a length mat with the infant positioned in a recumbent position. One person held the infant's head against the top of the length mat, while the second person held the infant's feet perpendicularly to the length mat and obtained the recumbent measurement.

### Exposures of interest

Maternal HIV status and concentrations of maternal estradiol, SHBG, progesterone, cortisol, GH1, IGF-1, and IGFBP-1 between 24- to 29-weeks' gestation were the primary exposures of interest. In a secondary analysis restricted to pregnant women with HIV only, the exposure of interest was duration of ART during pregnancy.

### Other covariates of interest

Data on socio-demographics, maternal anthropometrics, HIV immune status and ART history, including timing of ART initiation (prior to conception vs. during pregnancy), as well as newborn gestational age, feeding method, and duration of exclusive and total breastfeeding were collected. A second or third trimester ultrasound was performed by trained study staff to assess gestational age with an algorithm that considered ultrasound fetal measurements and maternal reported last menstrual period to assign the pregnancy's gestational age according the guidelines from the American College of Obstetricians and Gynecologists, the American Institute of Ultrasound in Medicine, and the Society for Maternal-Fetal Medicine (31). Socio-demographic information, obstetric history, and infant feeding data were collected via maternal self-report.

### Statistical analysis

Characteristics of pregnant women and infants were compared using Wilcoxon rank-sum and Chi-squared or Fisher Exact tests as appropriate. INTERGROWTH-21st reference standards at birth and the World Health Organization Anthro Survey Analyzer were used to calculate WAZ and LAZ at birth and one-year of life (32, 33).

Concentrations of analytes were log_10_-transformed to approximate a normal distribution and differences by HIV status were compared using Student's *t*-test. Generalized linear regression models with robust standard errors were fit to estimate mean differences by maternal HIV status for each systemic hormonal biomarker, unadjusted and adjusted for maternal age, maternal BMI, and gestational week of sample collection. In a secondary analysis restricted to women with HIV only, analytes were evaluated for differences by duration of ART during pregnancy, unadjusted and adjusted for the same covariates. To evaluate for associations between each hormone biomarker (log-transformed) with infant birth and 1-year WAZ and LAZ, generalized linear models with robust errors were fit, unadjusted and adjusted for maternal age and maternal BMI for the model of infant WAZ, maternal age and maternal height for the model of infant LAZ. For the analyses of 1-year WAZ and LAZ we also adjusted for weeks of exclusive breastfeeding and either birth WAZ or birth LAZ respectively. An interaction term between maternal HIV status and each hormone biomarker was introduced in each these models to assess effect modification by maternal HIV status.

Statistical significance was defined as a *p*-value ≤ 0.05, with use of 95% confidence intervals (CI) to provide the range of plausible values. Analyses comparing maternal and infant characteristics by HIV status were conducted using SAS Version 9.4 (SAS Institute Inc, Cary, NC, USA). Modeling analyses were conducted using STATA version 13 (StataCorp LP, College Station, TX, USA), and graphical plots were created using GraphPad Prism version 8.2 (GraphPad Software LLC, Boston, MA, USA).

## 3 Results

A total of 114 pregnant women, of which 46 (40%) were living with HIV, were included in this analysis. Two participants experienced a stillbirth, and 13 participants left the study before the birth visit was conducted, resulting in 99 infants who contributed anthropometric data, including 42 infants are HIV-exposed uninfected (HEU) and 57 HIV-unexposed uninfected (HUU). Pregnant women with HIV were older (27.4 vs. 25.8 years), had higher gravidity (3 vs. 1), and were less likely to breastfeed (73.8% vs. 100%) than women without HIV (Table 1). Among women with HIV, median CD4 count at enrollment was 494 cells/mm^3%^ and 90% had an enrollment HIV RNA level <40 copies/ml. The median gestational age at birth was 39.5 weeks [Interquartile Range (IQR) 37.7, 40.1] and did not differ by maternal HIV status. Fourteen (14.2%) infants were born prior to 37 weeks' gestational age, or preterm, including 6 (14.3%) infants born HEU. Infants who were HEU had a longer duration of exclusive breastfeeding at 17.4 weeks (IQR 4.8, 26.0 weeks) compared to 11.7 weeks for infants born HUU (IQR 7.6, 21.4 weeks).

**Table 1:** Maternal and infant characteristics at enrollment.

Characteristic	Women with HIV (*N* = 46)	Women without HIV (*N* = 68)	*P*-value
MATERNAL			
Age (years)	27.4 (24.0, 31.1)	25.8 (21.6, 29.4)	0.03
Marital Status^a^			0.003
Married	4 (8.6%)	8 (11.9%)	
Co-habiting	21 (45.7%)	11 (16.4%)	
Single	21 (45.7%)	48 (71.7%)	
Highest Educational Achievement			0.01
None/Primary School	2 (4.3%)	1 (1.5%)	
Junior Secondary/Senior Secondary	40 (87.0%)	47 (69.1%)	
Tertiary	4 (8.7%)	20 (29.4%)	
Gravidity	3 (2, 3)	1 (1, 3)	<0.001
Gestational Age at Maternal Lab Blood Draw (weeks)	26.9 (25.0, 28.0)	26.9 (25.4, 28.2)	0.43
Duration of Dolutegravir-based ART at Maternal Lab Blood Draw (weeks)	12.1 (8.7, 16.1)	NA	NA
Body Mass Index (kg/m^2^)^b^	26.6 (23.3, 32.4)	25.6 (23.0, 30.3)	0.52
HIV RNA <40 copies/ml^a^	32 (89.5%)	NA	NA
HIV RNA level (copies/ml)^c^	583 (55, 10,428)	NA	NA
Duration of Dolutegravir-based ART in Pregnancy (weeks)	24.6 (19.1, 28.3)	NA	NA
CD4+ Cell Count at Enrollment (cells/mm^3^)^a^	494 (352, 584)	NA	NA
	Infants HIV-Exposed Uninfected (*N* = 42)	Infant HIV-Unexposed Uninfected (*N* = 57)	
INFANTS			
Male	20 (47.6%)	21 (36.8%)	0.31
Gestational Age at Birth (weeks)	39.0 (37.6, 40.0)	39.6 (38.6, 40.2)	0.24
Preterm (<37 weeks gestational age)	6 (14.3%)	8 (14.3%)	1.00
Small for Gestational Age^d^	8 (20.0%)	6 (11.1%)	0.26
Ever Breastfeed^e^	31 (73.8%)	53 (100%)	<0.001
Duration of Exclusive Breastfeeding (weeks)	17.4 (4.8, 26.0)	11.7 (7.6, 21.4)	0.34
Infant Anthropometric Z-scores at Birth^f^			
Weight-for-gestational age Z-score	−0.26 (−1.17, + 0.95)	+0.03 (−0.96, + 0.48)	0.72
Length-for-gestational age Z-score	+1.12 (+0.47, + 2,06)	+1.14 (−0.10, + 2.67)	0.72
Infant Anthropometric Z-Scores at 1 Year of Life^g^			
Weight-for-age Z-score	+0.41 (−0.46, + 1.09)	+0.19 (−0.51, + 0.57)	0.32
Length-for-age Z-score	0.00 (−0.83, + 0.97)	+0.03 (−0.62, + 0.54)	0.91

Continuous variables are reported as medians with the 25th and 75th interquartile ranges, and a *p*-value from a Wilcoxon rank-sum test is reported. Categorical variables are reported as counts with percentages, and a *p*-value from a Fisher's exact test is reported.

ART, antiretroviral treatment.

^a^ Missing Data: One HIV-seronegative woman missing marital status; Eight women with missing viral load at enrollment; Nine women with missing CD4 at enrollment.

^b^ Body Mass Index calculated based on weight obtained at 1 month postpartum.

^c^ Median HIV-1 viral load for women who had a detectable HIV-1 viral load.

^d^ Small for gestational age defined as weight at birth < 10th percentile for gestational age.

^e^ Five infants were not seen after the birth visit and breastfeeding status is unknown.

^f^ INTERGROWTH-21st used to calculate anthropometric Z-scores at birth.

^g^ World Health Organization Anthro Software used to calculated anthropometric Z-scores at 1 year of life.

Median concentrations of bioavailable estradiol, cortisol, and free IGF-1 between 24 and 29 weeks' gestation were significantly lower, while the median IGFBP-1 concentration was significantly higher, among pregnant women with HIV compared to concentrations from women without HIV (Table 2). Upon log transformation of the assayed analytes, mean concentrations of bioavailable estradiol, cortisol, and free IGF-1 remained significantly lower among pregnant women with HIV compared to women without HIV. IGFBP-1, progesterone, and GH1 did not differ significantly between the two groups (Table 2).

**Table 2:** Analyte levels in gestational week 24–29 maternal plasma by maternal HIV status.

	Women with HIV *n* = 46^a^	Women without HIV *n* = 68^a^	*P* value^b^^,^^c^
Analyte: Median (IQR)			
Progesterone (ng/ml)	68.3 [47.0, 92.8]	80.5 [58.5, 100]	0.08
Estradiol (pg/ml)	6,885 [4,525, 9,571]	8,050 [6,110, 11,853]	0.14
Bioavailable estradiol (pg/mL)	442 [350, 778]	598 [452, 896]	0.006
Cortisol (pg/mL)	209 [161, 261]	280 [216, 342]	0.0003
SHBG (nmol/L)	781 [677, 920]	727 [534, 961]	0.20
Free IGF-1 (pg/mL)	35.0 [10, 234]	172 [57.5, 392]	0.006
IGFBP-1 (ng/mL)	246 [166, 379]	181 [115, 305]	0.05
GH1 (pg/mL)	610 [131, 1096]	263 [53.6, 1119]	0.21
Log_10_-Transformed Values Mean ± SD			
Progesterone	4.20 (0.45)	4.35 (0.45)	0.06
Estradiol	8.83 (0.51)	8.98 (0.56)	0.20
Bioavailable estradiol	6.21 (0.54)	6.47 (0.51)	0.01
Cortisol	5.34 (0.39)	5.61 (0.37)	0.0003
SHBG	6.67 (0.30)	6.53 (0.44)	0.06
Free IGF-1	4.04 (1.69)	4.91 (1.48)	0.005
IGFBP-1	5.41 (0.84)	5.22 (0.68)	0.19
GH1	5.97 (1.62)	5.27 (2.35)	0.08

NB: All women had a BMI ≤ 40 kg/m^2.^

GH1, Growth Hormone 1; IGF-1, Insulin-like Growth Factor-1; IGFBP, Insulin-like Growth Factor Binding Protein; SHBG, Sex-Hormone Binding Globulin.

^a^ *N* = 66 for the HIV- group for IGF-1 and IGFBP-1. *N* = 45 for estradiol, progesterone, cortisol, and bioavailable estradiol, and *N* = 44 for IGF-1 and IGFBP-1 for the women with HIV group.

^b^ Raw data values were analyzed using the Wilcox rank-sum test.

^c^ Log-transformed values were analyzed by Student *t*-test with equal variance.

**Figure 1:**
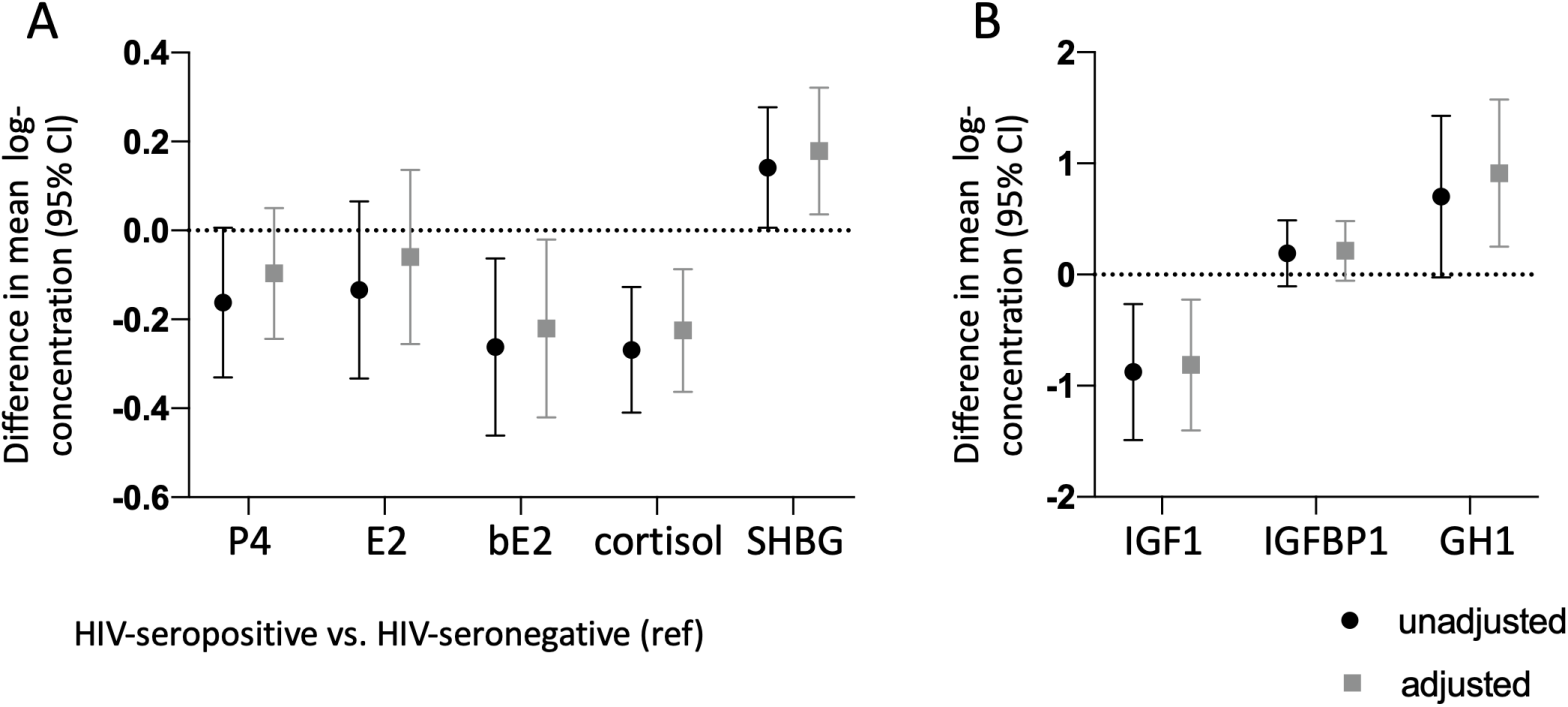
Lower concentrations of bioavailable estradiol, cortisol, and IGF-1, and higher concentrations of GH1 and IGFBP-1 in pregnant women with HIV vs. pregnant women without HIV. Maternal progesterone (P4), estradiol (E2), bioavailable estradiol (bE2), cortisol, and sex-hormone binding globulin (SHBG) are shown in **(A)** Maternal insulin-like growth factor-1 (IGF-1), insulin-like growth factor binding protein-1 (IGFBP-1), and pituitary growth hormone 1 (GH1) are shown in **(B)** All analytes were measured between gestational week 24–29. Difference in mean from the HIV-seronegative group (indicated by the dotted line), with 95% confidence interval, are shown for the HIV-seropositive group unadjusted (black symbols) and adjusted (gray symbols) for maternal age, maternal BMI, and gestational age at the time of sample collection. All data are log-transformed.

After adjusting for maternal BMI, maternal age, and gestational age at the time specimen collection, mean log-transformed concentrations of bioavailable estradiol, cortisol, SHBG, free IGF-1, and GH1 were significantly different between groups, with pregnant women with HIV having lower bioavailable estradiol {adjusted difference −0.22 [95% Confidence Interval (CI): −0.42, −0.02], *p* = 0.031}, cortisol [adjusted difference −0.22 (95% CI: −0.36, −0.09), *p* = 0.001], and free IGF-1 [adjusted difference −0.81 (95% CI: −1.40, −0.22) *p* = 0.007], but higher SHBG [adjusted difference 0.18 (95% CI: 0.04, 0.32), *p* = 0.014], and GH1 [adjusted difference 0.91 (95% CI: 0.25, 1.58), *p* = 0.007] (Figure 1). Duration on ART in pregnancy among women with HIV was not significantly associated with any of the analytes after adjusting for maternal age, maternal BMI, and gestational week of sample collection, although a trend for a negative association was observed with GH1 (β: −0.006, *p* = 0.06).

Overall, analyte concentrations did not vary by the sex of the fetus. However, in analyses stratified by maternal HIV status, female fetal sex was associated with lower levels of log-transformed free IGF-1 [mean difference −1.34 (95% CI: −2.3, −0.38), *p* = 0.006] and higher levels of IGFBP-1 [mean difference 0.56 (95% CI: 0.07, 1.04) *p* = 0.024] only among pregnancies of women with HIV. No associations between fetal sex and any of the analytes were observed in women without HIV.

**Figure 2:**
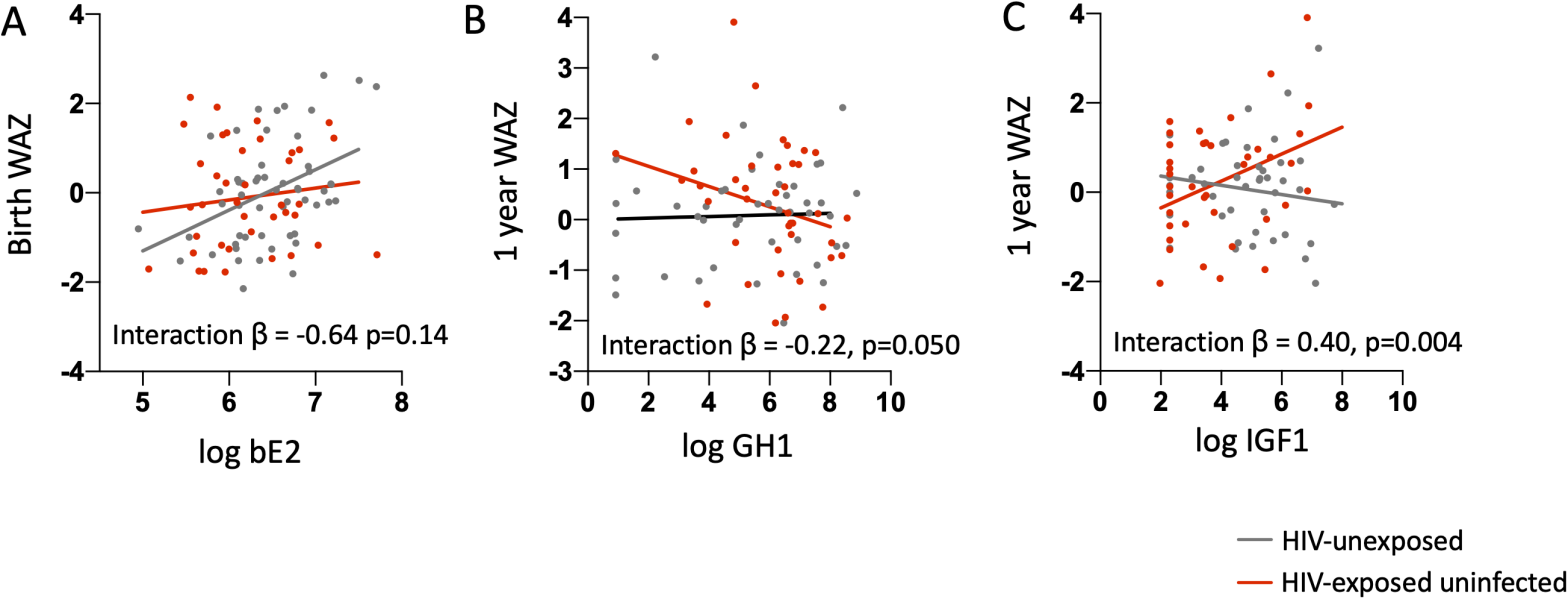
Associations between maternal mid-gestation hormone and growth factor concentrations and infant birth and 1-year weight for age z-score. **(A)** Log-transformed maternal bioavailable estradiol (bE2) levels are plotted against birth weight-for-age Z-score (birth WAZ) for infants who are HIV-exposed uninfected (red dots) and HIV-unexposed (grey dots). Linear regression lines by HIV exposure status based on a model that included an interaction term for HIV exposure status and log maternal bE2 and was adjusted for maternal age and maternal BMI are shown. The association between maternal bE2 and birth WAZ is only significant for the HIV-unexposed group. **(B)** Log-transformed maternal growth hormone 1 (GH1) and **(C)** insulin-like growth factor-1 (IGF-1) levels are plotted against weight-for-age Z-score (WAZ) at 1 year of age for infants HIV-exposed uninfected (red dots) and HIV-unexposed (grey dots). Linear regression lines by HIV-exposure status based on a model that included an interaction term for HIV status and log GH1 or log IGF-1 and adjusted for maternal age, maternal BMI, length of exclusive breastfeeding, and birth WAZ are shown. The association between GH1 or IGF-1 and 1-year WAZ is only significant for the HIV-exposed group.

The only hormone significantly associated with birth WAZ was bioavailable estradiol, with higher log-transformed bioavailable estradiol concentrations associated with higher birth WAZ after adjusting for maternal age and BMI [β: 0.62 (95% CI: 0.17, 1.06); *p*-value = 0.006]. When stratified by maternal HIV status, the association between birth WAZ and bioavailable estradiol was only significant among the infants who were HUU, but not for infants who were HEU. Introduction of an interaction term for maternal HIV status and log bioavailable estradiol resulted in an interaction coefficient of −0.64, which led to a flattening of the positive association between maternal estradiol and birth WAZ for the infants HEU. However, the *p*-value for the interaction term did not reach significance (*p*-value = 0.14) (Figure 2A, Table 3).

**Table 3:** Modeling results for each log-transformed analyte as predictor for birth WAZ, including an interaction term of log-transformed analyte and maternal HIV status and adjusting for maternal age and maternal BMI.

Coefficient (95%CI)	Progesterone Model	Estradiol Model	Bioavailable estradiol Model	Cortisol Model	SHBG Model	IGF-1 Model	IGFBP-1 Model	GH1 Model
Analyte (log transformed)	0.76 (−0.056, 1.57)	0.84 (0.37, 1.30)	0.91 (0.41, 1.40)	0.66 (−0.24, 1.55)	0.16 (−0.51, 0.83)	0.14 (−0.06, 0.34)	−0.16 (−0.59, 0.27)	−0.06 (−0.21, 0.10)
Maternal HIV status	3.56 (−1.18, 8.3)	3.31 (−4.22, 10.8)	4.07 (−1.31, 9.4)	5.25 (−2.18, 12.7)	−1.53 (−10.9, 7.8)	0.41 (−0.98, 1.81)	−0.91 (−4.8, 3.0)	−0.55 (−2.3, 1.2)
Analyte*maternal HIV status Interaction term (*p*-value)	−0.87 *p* = 0.14	−0.38 *p* = 0.38	−0.64 *p* = 0.14	−0.97 *p* = 0.16	0.21 *p* = 0.77	−0.11 *p* = 0.48	0.14 *p* = 0.69	0.08 *p* = 0.58
Maternal age	0.011 (−0.32, 0.54)	0.019 (−0.27, 0.06)	0.011 (−0.03, 0.06)	0.00 (−0.04, 0.04)	0.00 (−0.04, 0.05)	0.01 (−0.03, 0.05)	0.01 (−0.04, 0.05)	0.00 (−0.04, 0.04)
Maternal BMI	0.043 (−0.01, 0.96)	0.05 (−0.00, 0.10)	0.037 (−0.013, 0.09)	0.048 (−0.00, 0.10)	0.045 (−0.01, 0.10)	0.032 (−0.02, 0.08)	0.03 (−0.02, 0.09)	0.04 (−0.02, 0.09)

GH1, growth hormone 1; IGF−1, insulin-like growth factor-1; IGFBP, insulin-like growth factor.

The relationships between GH1 and free IGF-1 with infant WAZ at one year of life were significantly modified by maternal HIV status after adjusting for maternal age, BMI, weeks of exclusive breastfeeding from birth, and infant birth WAZ [*β* for GH1*maternal HIV status interaction term: −0.22; *p* = 0.005] and for IGF-1*maternal HIV status interaction term: 0.40; *p* = 0.004] (Figures 2B, C, Table 4). Associations of maternal GH1 and free IGF-1 with infant WAZ at 1 year of life were in opposite directions for infants who were HEU, compared to having no association with WAZ at 1 year of life among infants who were HUU. For infants who were HEU, higher gestational exposure to GH1 and lower IGF1 concentrations were associated with lower WAZ at 1 year of life.

**Table 4:** Modeling results for each log-transformed analyte as predictor for infant WAZ at 12 months of age, including an interaction term of log-transformed analyte and maternal HIV status and adjusting for maternal age, maternal BMI, breastfeeding, and birth WAZ.

Coefficient (95%CI)	Progesterone Model	Estradiol Model	Bioavailable estradiol Model	Cortisol Model	SHBG Model	IGF-1 Model	IGFBP-1 Model	GH1 Model
Analyte (log transformed)	−0.10 (−0.88, 0.67)	−0.22 (−0.79, 0.35)	−0.23 (−0.73, 0.26)	−0.33 (−1.1, 0.43)	0.065 (−0.54, 0.67)	−0.10 (−0.28, 0.07)	0.098 (−0.34, 0.53)	0.016 (−0.12, 0.15)
Maternal HIV status	0.59 (−3.6, 4.8)	3.9 (−3.1, 10.9)	1.67 (−2.7, 6.0)	−1.27 (−8.2, 5.8)	1.63 (−8.4, 11.7)	−1.52 (−2.7, −0.32)	2.4 (−1.2, 5.9)	1.46 (0.04, 2.9)
Analyte*maternal HIV status Interaction term (*p*-value)	−0.11 *p* = 0.84	−0.42 *p* = 0.29	−0.25 *p* = 0.48	0.25 *p* = 0.70	−0.22 *p* = 0.77	0.40 *p* = 0.004	−0.41 *p* = 0.21	−0.22 *p* = 0.05
Maternal age	0.015 (−0.02, 0.05)	0.010 (−0.03, 0.05)	0.012 (−0.03, 0.05)	0.016 (−0.03, 0.06)	0.19 (−0.02, 0.06)	0.017 (−0.02, 0.05)	0.017 (−0.02, 0.06)	0.019 (−0.02, 0.05)
Maternal BMI	0.013 (−0.03, 0.06)	0.004 (−0.04, 0.05)	0.011 (−0.03, 0.05)	0.013 (−0.03, 0.06)	0.014 (−0.03, 0.06)	0.017 (−0.02, 0.06)	0.01 (−0.04, 0.06)	0.00 (−0.02, 0.06)
Weeks exclusive breastfeeding	−0.025 (−0.05, 0.00)	−0.021 (−0.04, 0.00)	−0.022 (−0.05, 0.00)	−0.025 (−0.05, −0.0)	−0.026 (−0.05, −0.0)	−0.023 (−0.05, 0.00)	−0.019 (−0.04, 0.00)	−0.023 (−0.05, 0.00)
Birth WAZ	0.35 (0.12, 0.58)	0.40 (0.15, 0.64)	0.39 (0.14, 0.63)	0.36 (0.12, 0.59)	0.35 (0.11, 0.59)	0.39 (0.16, 0.63)	0.38 (0.14, 0.62)	0.36 (0.13, 0.60)

GH1, growth hormone 1; IGF-1, insulin-like growth factor-1; IGFBP, insulin-like growth factor binding protein; SHBG, sex-hormone binding globulin; BMI, body mass index; WAZ, weight for age z-score.

No significant associations were noted between analytes and infant LAZ at birth or at one-year of life.

## 4 Discussion

In this cohort of pregnant women participating in the Botswana-based Tshilo Dikotla study, women with HIV receiving DTG-based ART in pregnancy had alterations in hormone and growth factor levels between 24 and 29 weeks of pregnancy, including lower concentrations of cortisol, bioavailable estradiol, and IGF-1, and higher concentrations of pituitary-derived GH1 compared to pregnant women without HIV. Our previous work has demonstrated lower mid-gestation progesterone, higher estradiol, and similar cortisol levels among pregnant women with HIV receiving protease inhibitor (PI)-based ART compared to those receiving non-PI-containing regimens and women without HIV (24, 25), suggesting that some of the effects on, at least, estradiol and cortisol may be due to the type of ART rather than due to HIV infection. This is the first study to evaluate mid-gestation hormone levels in a cohort of pregnant women on DTG-based ART, a currently recommended first-line regimen in the majority of settings.

Cortisol, bioavailable estradiol, and IGF-1 have previously been associated with fetal growth (17, 19, 22, 34). Our findings of a positive relationship between maternal bioavailable estradiol and infant birth weight among pregnant women without HIV and their infants are consistent with published studies (35, 36). However, maternal HIV status modified this relationship, dampening the positive relationship between bioavailable estradiol and fetal growth. Our findings suggest that fetal exposure to increasing bioavailable estradiol concentrations in the presence of maternal HIV infection and exposure to a DTG-based ART regimen did not yield increased fetal growth. While only 3 women were on ART from conception, the median duration of use in pregnancy of the DTG-based ART regimen was 24.6 weeks, likely explaining the fact that 89.5% of women were virally suppressed to <40 copies/ml by time of enrollment into the Tshilo Dikotla study. While identifying the contributions of fetal exposure to HIV-1 virus vs. the maternal ART regimen is not possible, further studies are needed to understand the mechanisms underlying the association between bioavailable estradiol and fetal growth.

Cortisol levels were significantly lower in women with HIV in our study. This is in contrast to previous observations of similar cortisol levels between women with HIV on protease inhibitor-based ART and women without HIV (25), suggesting an association between DTG-based ART and lower cortisol levels. While we did not observe an association between cortisol and birth weight, hypercortisolism is expected in pregnancy and contributes to the physiologic maternal insulin resistance that facilitates mobilization of maternal nutrients for fetal growth (20, 37). Cortisol production is driven by placental derived corticotropin-releasing hormone, so the lower cortisol levels we observed in women with HIV may be an indication of placental dysfunction (37). Another indicator of placental dysfunction is our observation of higher pituitary GH1 levels in women with HIV. A healthy placenta is responsible for producing placental GH2 with concentrations rising to sufficient levels by the 24th to 29th week of pregnancy, resulting in suppression of pituitary stimulated production of GH1 (22). Thus, deficient placental production of GH2 may be responsible for the higher GH1 levels we observed in women with HIV. Future studies investigating both GH2 and corticotropin-releasing hormone levels in pregnant women with HIV, as well as studies directly investigating placental dysfunction, are merited.

Interestingly, while we observed no relationship between any of the analytes measured and overall infant WAZ at 1 year of life, maternal HIV status modified the relationship between GH1 and IGF-1 and 1-year WAZ. No associations were seen between GH1 or IGF-1 and WAZ at 1 year in infants who were HUU. However, among infants who were HEU, fetal exposure to higher maternal mid-gestation GH1 and lower maternal mid-gestation IGF-1 was associated with lower infant WAZ at 1 year of life. The placental GH2-IGF-1 axis plays a role in fetal growth and associations with birth weight have been shown (38). While we were unable to measure placental GH2, higher concentrations of pituitary-derived GH1 found in women with HIV in our study may suggest placental dysfunction and a likely perturbation in the GH2-IGF-1 axis. However, the association noted between concentrations of IGF-1 with infant WAZ at 1 year of life in the infants HEU in our study is less clear. One possibility may relate to IGF-1 levels in breast milk, as higher levels of breast-milk IGF-1 have been shown to correlate with higher infant weight at 13 months (39). If the lower IGF-1 levels that we observed in mid-gestation in women with HIV extend to lower IGF-1 in breast milk, then this may influence infant growth in the first year. However, we did not collect breastmilk and thus were unable to quantify IGF-1 levels. Further work is needed to assess IGF-1 levels in breastmilk by maternal HIV status and associations with infant growth.

The clinical significance of the observed associations between mid-gestation maternal hormones and growth factors and fetal, as well as infant anthropometrics, will require validation in future studies. The Development Origins of Health and Disease (DOHaD), proposed by David Barker as a conceptual framework to understand how the prenatal period programs for adult health and disease (40, 41), hypothesizes that gestational mechanisms that limit fetal growth result in fetal adaptations in physiology and metabolism that prompt fetal epigenetic changes ultimately associated with increased risk of cardiovascular disease and diabetes as a child advances into adulthood (42). Therefore, expanding the design of future studies to include analyses of epigenetic modifications and associated transcriptional changes in the presence of altered maternal hormones and growth factors is of importance.

Our study was limited by its small sample size and inability to explore associations between different ART regimens and hormone and growth factor concentrations, as all women with HIV in our study were on the same ART regimen. Nonetheless, our work represents one of the largest studies evaluating pregnancy hormones and growth factors in pregnant women with HIV on newer antiretrovirals such as DTG. We acknowledge that some hormone concentrations, such as cortisol and GH1, fluctuate by time of day. While all blood draws were performed between 7 am and noon, we cannot exclude the fact that some variation may have been due to the timing of the blood draw. We also recognize that abstraction of birth weight and length from medical records may reflect some inaccuracies compared to the practice of obtaining three sequential measures by trained study staff, a practice that was possible when infants were brought to their one-year study visit, but was not feasible at birth.

In summary, levels of select maternal systemic hormones and growth factors associated with fetal growth were found to be significantly different between pregnant women living with HIV receiving DTG-based ART and women without HIV. These differences were associated with lower WAZ-scores at 1 year in infants HIV-exposed uninfected, but not in infants born to women without HIV. Given our prior work demonstrating differences in maternal hormones in pregnancy by antiretroviral drug class (24–27), it would be important to conduct further research comparing hormones and growth factors in pregnancy among drug classes to identify ART regimens that optimize pregnancy outcomes, including fetal growth. It would also be valuable to incorporate surrogate measurements of placental health in future work with a goal of identifying modifiable biological pathways to ensure that all infants who are HIV- and ART-exposed achieve good fetal and postnatal growth.

## Data Availability

The raw data supporting the conclusions of this article will be made available by the authors upon reasonable request.
